# Technology Complements Physical Examination and Facilitates Skills Development among Health Sciences Clerkship Students: *An Integrative Literature Review*

**DOI:** 10.5334/pme.903

**Published:** 2023-04-04

**Authors:** J. Lees, M. Bearman, T. Risor, L. Sweet

**Affiliations:** 1 Deakin University, AU; 2 University of Copenhagen, DK

## Abstract

**Background:**

Technology is increasingly present in the clinical environment. There is a dearth of investigation of the relationship between technology and touch concerning student learning of physical examination practices.

**Method:**

Integrative review methods were used to synthesise empirical literature to provide a comprehensive understanding of the relationship between physical examination, learning and technology in the context of health professional student clerkships.

**Results:**

Three databases including MEDLINE, CINAHL and Eric were searched for all articles published from 2014 to 2021 using terms relating to (i) physical examination, (ii) technology, and (iii) student clerkships. Thirty-three studies met the inclusion criteria. From the analysis, it is evident that technologies that intersect with learning of physical examination may broadly be apportioned into two categories: 1) technologies that mediate physical examination practices; and 2) technologies that mediate the learning of physical examination.

**Conclusions:**

This review indicates that technologies may have multiple roles in the student learning of physical examination, including technology mediating increased diagnostic accuracy and access to supplementary learning material relating to physical examination that is integrated for the clinical clerkship environment. It highlights a need to further understand the touch versus technology relationship and explore the dynamic intersection.

## Introduction

Physical examination has long been considered a cornerstone of clinical practice for healthcare professionals. However, with health services becoming increasingly mediated by technology, the prevalence of physical examination in clinical practice has declined, and consequently, it is surmised that there has also been a decrease in the teaching of touch-based physical examination [1,2,3]. Given the supposed influence of technology on physical examination, it is timely to investigate the relationship between physical examination, learning, and technology.

The recent necessity of social distancing due to the COVID-19 pandemic has reduced the clinical opportunity for student learning of touch-based physical examination [4,5]. While touch is a key component of the physical examination, learning touch offers more than diagnostic and treatment benefits, but also represents a sense of care, comfort, and non-verbal communication [6,7]. The pandemic alone cannot be held accountable for recent scrutiny of the learning of physical examination practices, as there has been wide discussion in the literature proposing that technology is interfering with physical examination and touch, and that there is growing reliance on technology [8,9]. There is increasing use of digital technology in clinical practice [1], but will this technology influence how students learn touch-based physical examination?

The discourse regarding touch and technology in the health professions is rather polarised. The dominant discourse presents technology, particularly as it relates to ‘cutting edge’ technology, with a techno-utopian lens [10], and even suggests that emerging artificial intelligence (AI) technologies may replace skills formerly performed by health practitioners [11]. In contrast, the call to find a “road back to the bedside” (p 1672) [12] or to “preserve the human touch in medicine in a digital age” (p E622) [13] preferences touch over technology. The literature suggests that teaching practices may be influenced with reduced frequency of physical examination learning opportunities for students [14,15,16,17], and there is concern regarding reduced competence and confidence [1]. It is largely hypothesised that this perceived change is related to an increase in availability and use of technology [2,3], and technology is accused of being the cause of the ‘lost art’ of touch-based physical examination skills [1]. However, there is little empirical evidence available to confirm this perceived trend in health student learning and scarce discussion regarding the intersection between technology and student learning of physical examination. Overall, it is unclear if embodied learning is being overlooked with the increased focus on the use of technology.

Conversely, rather than technology competing with touch in physical examination, technologies may provide the potential to refocus attention in clinical education towards the human aspects of being a healthcare professional [18]. Greater efficiency may offer more time available to move beyond calculation-centred diagnostics towards a greater emphasis for the learner on connecting with each patient [18]. Conscious and strategic use of technology may create opportunities to move both clinicians and teaching back to the bedside. There is an argument that the current formal bedside curriculum is not representative of contemporary practice and that there is opportunity for incorporating technology in bedside teaching sessions for better alignment between teaching and practice [8].

The concern that advancing technology is encroaching on touch-based physical examination skills for future clinicians, has prompted a call for attention to the clinical teaching of these skills [8,19]. Technology, particularly relating to diagnostics, may be perceived as more accurate and therefore excessively preferenced over information gathering through a comprehensive touch-based physical examination [20]. This ready embrace of technology is at odds with the argument that a competent touch-based physical examination and a well-considered diagnostic hypothesis could lessen the burden of expensive technologies [21]. However, despite technological advances that have transformed diagnostic capabilities in healthcare, physical examination continues to be considered an essential capability and is the foundation of effective diagnosis [21].

A focus on the dichotomy between touch versus technology affords the opportunity to understand how physical examination and technology dynamically connect, and the potentially significant role of technology to mediate learning physical examination within the clinical environment. While there is agreement in the literature regarding the importance of students learning touch-based physical examination in clinical practice, currently little is known about how technology can mediate student learning of physical examination in practice. This integrative review will take a broad view on this and aim to explore the relationship between physical examination, teaching, and technology within the clinical environment.

## Methods

An integrative review approach allows for the inclusion of a diversity of methodologies, clinical environments, and health professional student populations. We used the framework outlined by Whittemore and Knafl (2005) [22] to guide the review and enabled the researchers to systematically review and critique the literature, map the findings, and then synthesise the interpretations. This approach helped us to recognise the influence of social constructs, allowing for interpretive analysis of findings and the emergence of a more fluid epistemology [23].

### Definitions

For the purposes of this review, physical examination is defined as an integrated assessment of a person in order to determine their state of health and wellbeing. Digital technology refers to any tool in the clinical environment that is used to collect, employ, or convey digital information.

### Search strategy

An initial comprehensive search was conducted in MEDLINE, from which the search terms were refined, and then the search was run across three databases: MEDLINE, Eric, and CINAHL. The search terms were selected to describe the four concepts of interest: physical examination, student, technology, and clinical clerkship (Supplementary Appendix A). Databases were chosen because of their focus on the health professions, education, nursing and allied health professions, and biomedicine. While we did not conduct a systematic review, we utilized this structured nature of the PRISMA guidelines to guide reporting [24].

We included primary research published between 2014–2021 as literature published during this period is likely to reflect contemporary clinical practice environments and current technology usage trends.

### Inclusion/exclusion

The review included studies focused on the role of technology in learning physical examination with student learners from all primary health professions and set in clinical clerkship environments. We defined primary health as professionals who may be first contact person in health system and subsequently responsible for physical examination and included medicine, nursing, midwifery, pharmacy, dentistry, and physiotherapy. For consistency, only primary research published in English was included, with secondary sources of data, such as other reviews, examined for relevant primary data sources.

Health professional education programs were defined as work-based clinical training in locations such as teaching hospitals and community health facilities. These experiences are commonly referred to as clinical clerkships, placements, or rotations. For this review, all learning of physical examination that was situated within the clerkship environment was included. These included bedside learning, simulation within the clinical clerkship setting and blended learning tools used within the clerkship environment. Studies set in skills workshop settings or simulation contexts that were situated on-campus or away from the clinical clerkship setting were excluded. Studies that focused on qualified clinicians rather than students, and studies focusing on alternative and complementary medicine students were excluded (Table 1).

**Table 1:** Integrative review inclusion and exclusion criteria.


INCLUSION	EXCLUSION

**Student learners**	Qualified health professional in further training

**Located at the site of the clinical clerkship**	Any physical examination learning that is located away from the site of the clinical clerkship, including on-campus skills laboratories or simulation

**Primary health professions**	Alternative and complementary medicine students

**English language**	


### Data analysis and synthesis

Data analysis was carried out through an interpretive paradigm. Data synthesis was performed using thematic analysis. To undertake effective analysis, Whittemore and Knalf (2005) outline that the data from primary sources are re-ordered, coded, characterised into themes, and brought together into a cohesive and integrated summation [22].

### Thematic analysis

**Figure 1:**
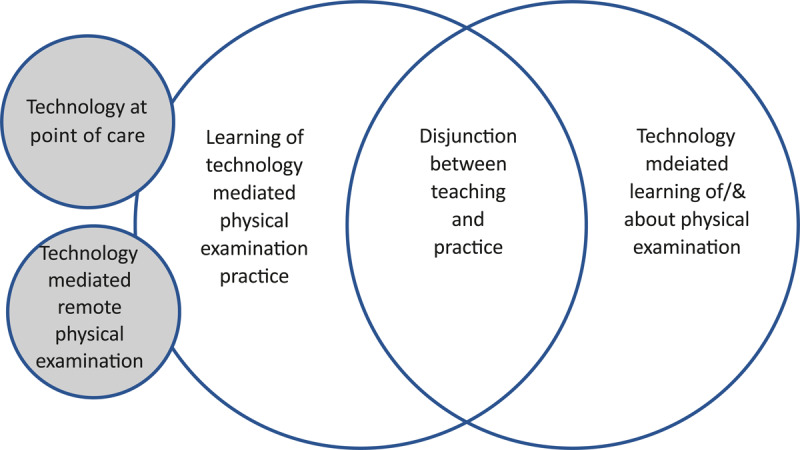
Visualisation of themes from the thematic analysis.

The thematic analysis was inductive and conducted by the primary researcher with discussion and refinement with the other researchers. The research team consisted of a physiotherapist, a physician, a midwife, and an educational specialist. Data analysis was represented visually to assist in the identification of patterns and relationships (Figure 1). Analysis suggested two major groups of studies. One group of studies described the relationships between technology and physical examination in practice, and the other group described the relationship between technology and learning of or about physical examination. Thirdly, there were two studies that did not fit either grouping but instead represented the disjunctions between health professional practice and education (See Table 2).

**Table 2:** Thematic analysis.


THEME	SUBTHEME	*CATEGORIES*	

Learning of technology mediated physical examination practice	Technology mediated remote Physical Examination	*Telemedicine* [37,38,39,40]	

	Technologies at the Point-of-Care	*Bedside Mobile Apps* [30,31,32,33]	

		*POCUS* [20,25,26,27,28,29]	

		*Infrared* [34]	

		*Smart Phone (general)*	[50,51,55]
	
Technology mediated learning of and about physical examination practice			[52,53,54]

		*App based blended learning* [44,45,46,47]	

		*Online Video library/E-module* [39,40,42,43]	

		*Simulation* [41,48,49]	

Disjunction between Education and Practice		[8,56]	


## Results

The initial search identified 243 studies and this list from further refined based on exclusion criteria and time limits to include 34 research studies (See Supplementary Appendix B for PRISMA diagram). These studies were summarised, and key findings were identified (Supplementary material C). Studies focused on a variety of health disciplines, including medicine (21), nursing (10), pharmacy (1), midwifery (1), and interprofessional (1), and were undertaken in the United States (12), Australia (4), Germany (3), Hong Kong (3), Canada (2), Ireland (2), Brazil (1), Bahrain (1), Colombia (1), Denmark (1), India (1), Japan (1), Taiwan(1), and the United Kingdom (1), The studies used different methods and methodologies, including ethnography, participant observation, qualitative surveys, quasi-experimental designs, and randomised control studies.

### Theme 1: Learning of technology-mediated physical examination practice

This theme represents studies that describe the learning of technology-mediated physical examination practice. We make the distinction here that all this technology use supports or enables clinical practice rather than directly benefits learning. There were two sub-themes: learning physical examination employing technologies at ‘point of care’; and technology-mediated remote physical examination.

### Technologies at the ‘point of care’

Sixteen studies described technologies that provided students with information at the bedside to assist in decision making and diagnosis and are used as a part of the physical examination process. Six reported on the use of smartphone devices in the clinical environment broadly, while five studies reported the use of Point of Care Ultrasound (POCUS): four on mobile applications (apps) at the bedside and one on infrared technology.

#### Point-of-care Ultrasound (POCUS)

Six studies examined the use of POCUS in clinical clerkships [20,25,26,27,28,29]. Three of these studies found that the inclusion of POCUS increased diagnostic accuracy when compared with traditional physical examination teaching [25,27,28]. For example, Mai, Woo [20] compared physical examination performed by a medical student that included POCUS, with physical examination performed by experienced vascular surgeons. In this study, students with the POCUS were highly accurate and more effective in detecting ascending abdominal aneurysms (AAAs) compared to surgeons using traditional physical examination [20]. Ho, Critchley [29] found that final-year medical students were readily able to perform transthoracic echocardiography with POCUS with minimal additional training during clinical clerkships [29].

#### Bedside apps

Smartphones are increasingly accepted at the bedside for the ready availability for quick access to reference checking, particularly using targeted apps. These apps are increasingly seen to be useful to assist in patient physical examination [30,31,32]. Kenny, Gaston [31] found that in a group of nursing students, access to mobile apps reduced student anxiety related to the performance of clinical skills at the bedside, including physical examination required for urinary catheterisation, nasogastric tube insertion and removal, enteral feeding, and wound care. Conversely, Bove and Carroll [33] found that app-based examination of blood pressure measurements was less accurate than a traditional examination with a blood pressure cuff.

#### Near-Infrared technology

One paper discussed the use of near-infrared technology to assist nursing students in doing physical examination of patients to locate a vein appropriate for cannulation [34]. However, this study did not find this technology as effective in assisting students for this purpose as it was for skilled clinicians, suggesting that the foundational palpation and site recognition skills were required prior to the addition of more advanced techniques.

### Technology-mediated remote physical examination

Five papers described technologies that mediated student interactions with patients for the purposes of remote clinical practice, including the performance of remote physical examination.

#### Telemedicine

Telemedicine clerkships are a novel approach providing an alternative to traditional in-person clerkships and allow for remote real-time physical examination utilising communication technologies. In parallel with the rapid practice shift for clinicians, the catalyst for developing remote clerkships for the studies included was a necessity, as social distancing measures precluded students from attending in person during the COVID-19 pandemic. These studies suggest that remote patient engagement may provide beneficial development of clinical practice, particularly in patient interviewing, case management, and communication for remote physical examination [35,36]. Telemedicine-based placements may provide an opportunity to develop skills that are likely essential competencies for clinicians into the future [35].

Various uses of telemedicine were explored to facilitate continuing practice development of students otherwise distanced from clinical environments. Chandra, Laoteppitaks [37] used telemedicine appointments for follow-up emergency department presentations. In Rupley et al.’s study [36], students worked in interprofessional teams to use telemedicine platforms to provide obstetric patients with antenatal and postpartum care, which included remote physical assessments. Weber, Dua [35] created an exclusively telemedicine-based four-week outpatient clinical placement for medical students, and in Cain et al.’s [38] study, medical students completed family medicine clerkships using a telemedicine platform. These clerkships all included remote physical assessment; however, evaluations of telemedicine placements identified that physical examination options were limited in this format [35,38]. This highlights the notion that telemedicine placements provide opportunities to learn a discrete set of non-touch-based examination skills required for remote platforms, rather than being an equivalent replacement for traditional clinical placements. The addition of monitoring equipment in patient homes for the measuring of vital signs assisted in overcoming some of the challenges of remote physical examination [35,36].

### Technology-mediated learning of, and about, physical examination practices

In contrast, to the above theme, which focused on technology mediated physical examination practices, this theme represents studies that describe technology-mediated learning of, and about, physical examination practices.

### Technology for blended learning

The technologies in the following categories were used in the clinical environment as an adjunct to patient encounters to facilitate learning about physical examination.

#### Online video repositories and e-modules

Student access to technology-based learning resources in the clinical environments was advantageous to facilitating learning physical examination [39,40,41,42,43]. In the study by Fog-Petersen, Borgnakke [39], a video repository was developed to supplement areas of practice that may have limited clinical opportunities during clerkships, such as mental status examination. This study found that the video library provided an effective explanation of the examination process, and students were readily able to transfer their experiences to the clinical setting. Lehmann et al. [40] also explored the use of video resources during clinical clerkships to learn the specialised performance of physical examination in paediatrics. The combination of the preparatory instructional videos, followed with bedside teaching, was felt to improve students’ achievement of pediatric physical examination competency.

The four papers in this sub-theme support authentic content as an important factor for learning experiences that are included during clinical clerkships for effective learning and student engagement [39,40,41,42,43]. Weiner [43] explored the use of an online module to support the learning of physical examination of chronic low back pain and found that this module significantly improved clinical skills in medical students evaluating chronic low back pain. Successful learning outcomes were also found by Tokunaga [42], who developed virtual physical assessment learning material to allow pharmacy students to understand the efficacy of drugs and assess for early signs of adverse effects through a virtual platform.

#### App-based blended learning

The use of mobile apps to provide students access to learning material related to physical examination during clerkships was explored by four studies [44,45,46,47]. Two studies conducted by S. O’Connor and Andrews [44,45] found the use of mobile apps to provide access to tailored educational material in clinical learning environments was strongly supported by students as it improved knowledge, confidence, and the performance of performance of PE, amongst other hands-on clinical skills. In a study conducted by Hsu et al. [46], a mobile app was developed to focus on physical examination as a support to nursing students during their clinical placement. It was found to have a positive influence on learning, particularly as the students could relate the content to their clinical context. Sonne et al. [47] explored the use of a clinical examination app for delivering blended learning to medical students during their internal medicine clerkships. They found that students demonstrated improved performance, which was perceived to be a result of the preparation in combination with clinical exposure.

#### Technology-mediated simulation

Three studies explored the use of simulation that was embedded into the clinical clerkship environment to support physical examination learning. Frequently, simulation is conducted on campus; in these examples, however, it was included as a blended approach alongside the clinical interactions. Angarita et al. [48] studied the use of simulation in combination with technology-based multimedia learning material to teach breast examination and found this method to be more effective than the traditional teaching approach of simulation exclusively. The study by Goldsworthy et al. [41] found that virtual auscultation using authentic real patient sounds (rather than synthetically derived sounds used by past interventions), had improved performance in nursing students’ competence in detecting cardiac murmurs. Pearson et al. [49] explored the implementation of simulation to provide a comprehensive basis for the learning of female pelvic examination that combined touch-based examination and transvaginal ultrasound. These high-fidelity simulations were shown to assist students to develop these physical examination skills that combined touch and ultrasound, and these skills were transferable to clinical encounters [49].

#### Smartphone general use for guiding practice and learning

Six studies highlighted the benefits of smartphones for timely access, to support decision-making, and for the provision of educational material in situ [50,51,52,53,54,55]. These studies are interpreted to be positioned as intersecting themes as demonstrated in the visual diagram (Figure 1). In these studies, smartphone use is explored more broadly to include usage to guide practice and learning. Students used their devices to gather information rapidly and this information facilitated learning about the physical examination topic while in the clinical environment. Collectively these studies found that smartphone usage was overall beneficial to clinical practice including physical examination [50,51,52,53,54,55].

Three studies recognised that there may be disparity between teaching practices and learning practices relating to usage of smartphones [52,53,54]. Gavali, Khismatrao [54] survey of medical students found that 88%, 99%, and 93% of first, second, and third-year medical students respectively, used their smartphones for “instant access” (p. 7) during bedside learning. This was independently driven rather than guided by clinical supervisors. Multiple studies similarly identified that the use of mobile devices at the bedside as a learning tool for physical examination was largely opportunistic rather than a formalised process [51,52,53,54].

### Disjunctions between education and practice

This theme was constructed from two observational studies and describes how technology leads to disjunctions between education and practice [8,56]. These observations suggested misalignments between learning and practice, pointing out insufficient engagement between both touch and technology, although the studies had very different findings. Bilello, Dubosh [56] conducted an evaluation of student’s physical examination practices during an emergency medicine clerkship using a high acuity chest pain case example and found that components of what would be considered a thorough physical examination practice were not consistently performed. The authors suggest that this disparity between best practice ideals and what was observed “may indicate a professional trend toward reliance on technology and laboratory diagnostic tools as a possible distraction to the power of a thorough physical examination” (p 586) [57]. This study highlighted that there is an awareness from both students and clinical educators of a decrease in physical examination proficiency, and that there “appears that the movement toward technology and away from the bedside caused physical examination skills to be lost” (p 4) [8].

A qualitative study of bedside physical examination teaching conducted by Rousseau, Könings [8] suggested that the current formal bedside curriculum is not representative of contemporary practice, and that incorporating technology in bedside teaching sessions would create better alignment between teaching and practice [8]. The teaching clinicians suggested that “removing skills and manoeuvres of limited use from the curriculum and suggested incorporating technology in bedside teaching sessions, such as ultrasound, videos, and photos” (p 7) [8]. Consequently, the authors suggest that new skills used in current physical examination practices, such as the use of POCUS, must be integrated into such a curriculum to stay current.

## Discussion

This review suggests that technology currently is given two major roles in students’ learning of physical examination during clinical clerkships: Students may experience the use of technology to mediate physical examination practice, and technology may be integrated into the clinical environment to mediate learning. The debate over touch versus technology often focuses on the central question of whether technology can replace the tactile experience of touch in physical examination. The findings of this review suggest that this polarization was not prominent in the literature, but rather the two primary uses of technology are balanced with hands-on experience. However, the potential for preferencing technology over touch-based physical examination did appear in the observational studies [8,56].

The findings support the integration of technology to mediate physical examination for student learners. These findings may be particularly relevant for administrators of clinical clerkship programs. The studies included suggest that students can become proficient users of technologies such as POCUS with minimal additional training and reliably access to resources at the bedside to guide physical examination and inform clinical decision-making. Opportunity exists for new skills such as these to be incorporated into the clinical physical examination curriculum to parallel contemporary practice. Our findings suggest that uptake of technologies such as POCUS is slow, and a gap exists between current clinical practice and what is included in the bedside curriculum [8]. Conceivably, a cultural preferencing of touch-based physical examination remains [1,3,12,14].

Clinical examination teachers may be interested in the findings that highlighted the use of technology to improve teaching. Examples of technology that is beneficially being assimilated into the clinical environment during clinical clerkships to aid student learning and strengthening the quality of clinical care [31,50] included video repositories, online resources, and mobile devices. The advantages of using ‘just-in-time’ technology for rapid information gathering to assist clinical decision-making or guidance for clinical skills prior to their execution were highlighted [31,51,53]. Facilitators of technology integration for learning of physical examination were most notably student preference for increased access to digital resources in the clinical environment, for self-directed learning, and for problem-solving in clinical practice. Conversely, resistance from senior clinicians was identified as a primary barrier [51,52,53]. It appears from the observational studies that there seems to be a disconnect between the skills clinicians are expected to demonstrate at the bedside as role-models, and the skills that are required and routinely used in daily practice [8]. Clinicians interviewed by Rousseau, Könings [8] suggest that this disparity may have contributed to the loss of some physical examination skills which are less commonly used in contemporary practice, potentially because of advancements in available diagnostic technologies. Physical examination curriculum may need to evolve to include new skills such as the use of ultrasonography [8].

The role of technology in the development of non-tactile skills was highlighted by the studies that considered remote telemedicine placements. There is still a need to further understand the intersection between touch and technology for information gathering in physical examination and clinical reasoning. It can be argued that physical examination and clinical reasoning are inextricably linked, particularly when considering the contemporary approach of the concept of hypothesis-driven physical examination [57]. Further exploration of the clinical reasoning processes in conjunction with the manual aspects of the physical examination may provide additional insights about how to select and prioritise touch-based and technology-mediated examinations. Taking this early experience into future clinical teaching, it could be inferred that strategic selection of these opportunities may be made to develop these communication and reasoning components of physical examination. This would also require conscious effort made to compensate for the touch-based aspects of physical examination that are difficult to learn in virtual settings. However, critique of learning physical examination centred on observation only was notably lacking. With a growing shift toward remote learning [58], this gap in the discussion highlighted the need to further understand what are the implications if a pattern emerges with future clerkships, including telemedicine in place of in-person clerkships.

## Strengths and Limitations of this review

Comprehensive search and data collection strategies were used, and the resulting synthesis provides an overview of current areas where technology and teaching of physical examination intersect. However, there are also limitations. This includes 1) a single reviewer completing the search and data collection, 2) the majority of studies concerned medical education, and 12 of 33 studies were located in the US, which may bias the findings and limit application, and 3) all articles retrieved were in English. Although not a fundamental component of integrative review methodology [22], study quality was not rigorously assessed. As noted, several studies included surveys post intervention and social desirability bias may influence respondents’ answers.

## Conclusion

This integrative review indicates that technologies can have numerous roles in students’ learning of physical examination. These technologies can be largely divided into those that mediate physical examination practice and those that mediate the learning of physical examination. This review highlights a need to understand the touch versus technology dichotomy and explore the dynamic intersection, particularly identifying the need for future studies to investigate student use of technologies in learning physical examination practice, and technologies that support student learning of touch and physical examination. Understanding the confluence between touch and technology is pertinent to identify that they are not in opposition, but rather the further understanding of how they may be incorporated in physical examination to offer the greatest benefit is required.

## Additional File

The additional file for this article can be found as follows:

10.5334/pme.903.s1 Supplementary files. Appendix A to C.
