# Supplementary material for: Technology Complements Physical Examination and Facilitates Skills Development among Health Sciences Clerkship Students: An Integrative Literature Review
Source: Perspect Med Educ. 2023 Apr 4;2(1):109–19. doi: 10.5334/pme.903 (PMC10077975; doi:10.5334/pme.903)
Supplement: Supplementary files. — Appendix A to C. [file pme-12-1-903-s1.pdf]

Supplementary Appendix A- Search terms

	MEDLINE (MeSH)	CINAHL	ERIC
Concept 1<br>Physical<br>Examination	TI "physical exam**" or AB "physical exam*"<br>TI "Professional touch" or AB "Professional touch"<br>TI palpation or AB palpation<br>MH Physical examination	TI "physical exam**" or AB "physical exam*"<br>TI "Professional touch" or AB "Professional touch"<br>TI palpation or AB palpation<br>MH Physical examination	TI "psychomotor skill*" or AB "psychomotor skill*"<br>TI "manual skill*" or AB "manual skill*"<br>TI touch* or AB touch*<br>TI palpation or AB palpation<br>TI "physical examination*" or AB "physical examination*"<br>TI "Professional touch" or AB "Professional touch"
Concept 2<br>Technology	TI technolog* or AB technolog*<br>TI digital technolog* or AB digital technolog*<br>TI Artificial intelligence or AB Artificial intelligence<br>MH Digital Technology	TI technology or AB technology<br>TI Artificial intelligence or AB Artificial intelligence<br>MH technology	TI technology or AB technology<br>TI Artificial intelligence or AB Artificial intelligence
Concept 3<br>Clinical placements	TI "Clinical placement*" or AB "Clinical placement*"<br>TI "teaching rounds" or AB "teaching rounds"<br>TI "situated learning" or AB "situated learning"<br>TI "clinical learning" or AB "clinical learning"<br>TI "clinical rotation" or AB "clinical rotation"	TI "Clinical placement*" or AB "Clinical placement*"<br>TI "teaching rounds" or AB "teaching rounds"<br>TI "situated learning" or AB "situated learning"<br>TI "clinical learning" or AB "clinical learning"	TI "Clinical placement*" or AB "Clinical placement*"<br>TI "teaching rounds" or AB "teaching rounds"<br>TI "situated learning" or AB "situated learning"<br>TI "clinical learning" or AB "clinical learning"<br>TI "clinical rotation" or

	TI "Clinical training*" or AB "Clinical training*"<br><br>TI "student placement" or AB "student placement"<br><br>TI internship or AB internship<br><br>MH Hospitals, Teaching<br><br>MH Teaching Rounds	TI "clinical rotation" or AB "clinical rotation"<br><br>TI "Clinical training*" or AB "Clinical training*"<br><br>TI "student placement" or AB "student placement"<br><br>TI internship or AB internship<br><br>MH student placement	AB "clinical rotation"<br><br>TI "Clinical training*" or AB "Clinical training*"<br><br>TI "student placement" or AB "student placement"<br><br>TI internship or AB internship
Concept 4<br><br>Student Learners	TI Student* or AB Student*<br><br>TI trainee* or AB trainee*<br><br>TI intern or AB intern<br><br>TI novice or AB novice<br><br>MH "Students, Health Occupations+"	TI Student* or AB Student*<br><br>TI trainee* or AB trainee*<br><br>TI intern or AB intern<br><br>TI novice or AB novice<br><br>MH Interns and Residents	TI Student* or AB Student*<br><br>TI trainee* or AB trainee*<br><br>TI intern or AB intern<br><br>TI novice or AB novice

Supplementary Appendix B *PRISMA Flow Diagram*

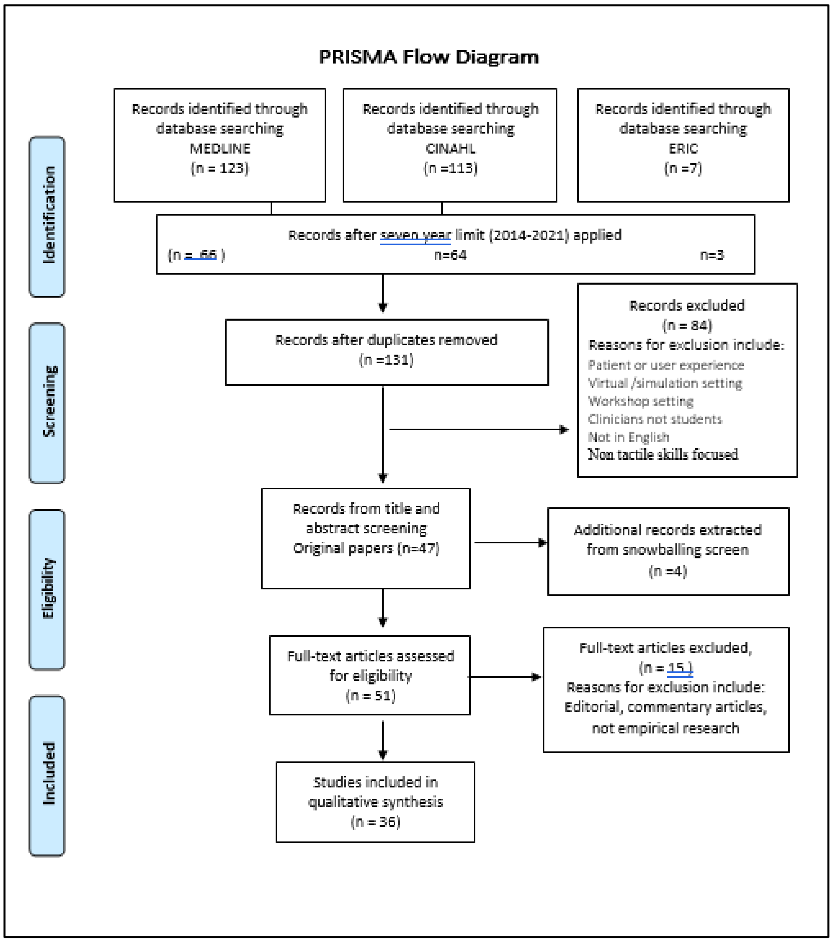

Supplementary Appendix C; Summary table*

	Author & Year	Title	Participants (sample + setting)	Methods	Aim	Key Findings related to physical examination skills learning	THEMES
1	Kenny, Gaston, Powers, and Isaac-Dockery (2020)	Anxiety in nursing students: The impact of using mobile technology with quick response codes.	Nursing;<br>42 yr1 students and 4 nursing educators;<br>**USA**	Quasi experimental design	1)To evaluate the effect of mobile device application for quick response codes on students' anxiety levels in the clinical setting.<br>2) To explore the clinical faculty experience with and perceptions of the technology.	- This technology provides access to resources pertinent to providing patient care<br>- Promote student self-directed learning and problem solving<br>- Reduce student stress.	Mobile Device Application<br>-Quick access at point of care<br>- Improve patient care<br>- Reduce student stress and increase self-efficacy
2	Yan et al. (2018)	Junior medical student performed focused cardiac ultrasound after brief training to detect significant valvular heart disease	Medicine; 10 yr3 students; **Hong Kong**	Comparative design	To teach medical students to perform FOCUS to detect valvular heart lesions	Students became proficient in detecting lesions with FOCUS<br>Student performed FOCUS was better than manual physical examination in detecting significant valvular lesions.	Point of care Ultrasound<br>Increased diagnostic accuracy
3	Bilello et al. (2018)	Fourth-year medical students do not perform a focused physical examination during a case-based simulation scenario	Medicine; 27 yr4 students;<br>**USA**	Prospective observational study	To determine the utility of a cardiac case-based simulation scenario to assess physical examination performance medical students during an emergency medicine clerkship.	Components of a focused physical examination were not uniformly performed in a high-acuity chest pain simulation scenario<br>These findings may indicate a professional trend toward reliance on technology and lab diagnostic tools as a possible distraction to the power of a	Observation of trends<br>Over reliance on Technology<br>Poor performance of PE<br>? Tech vs touch

						thorough physical examination	
4	Rousseau et al. (2018)	Overcoming the barriers of teaching physical examination at the bedside: more than just curriculum design.	Medicine; 21 teaching clinicians<br><br>**Canada**	Constructivist design	To explore residents' and physicians' perceptions of physical examinations teaching at the bedside following the implementation of a new structured bedside curriculum: What are the potentially persisting barriers and proposed solutions for improvement?	- The current formal bedside curriculum was not considered to be representative of the practice of a general internist<br><br>-Clinicians suggested removing skills of limited use from the curriculum and rather incorporate technology in bedside teaching sessions, such as ultrasound, videos and photos<br><br>- Appears that a movement towards/reliance on technology away from the bedside, contribute to lost ability to do physical exam to provide answers	Observation of trends<br><br>Over reliance on technology taking away from bedside<br><br>Current formal bedside curriculum blueprint was not considered to be representative<br><br>Suggested incorporating technology in bedside teaching sessions
5	Andersen et al. (2014)	Feasibility and accuracy of point-of-care pocket-size ultrasonography performed by medical students	Medicine; 30 final year students;<br><br>**USA**	Quantitative analysis	Aimed to investigate whether medical students with minimal training were able to successfully acquire and interpret ultrasound images using a pocket-size imaging device (PSID) as a supplement to their clinical practice	Medical students with minimal training were able to use PSID as a supplement to standard physical examination and successfully acquire acceptable relevant organ recordings for presentation and correctly interpret these with great accuracy	Point of care Ultrasound<br><br>Increased diagnostic accuracy
6	Sonne et al. (2021)	Significant differences in written assessments as a result of a blended	Medicine; 26 medical students<br><br>**Germany**	Random controlled study	To conduct a randomised controlled pilot study to see if an additional app-based blended-learning	An app-based blended-learning approach lead<br><br>-To improvement in test scores<br><br>-To more in-depth learning of	**Technology as an adjunct**<br><br>-Improvement in test scores

		learning approach used in a clinical examination course in internal medicine: a randomised controlled pilot study			strategy resulted in better student self-assessments and their ability to perform physical examinations	bedside examination skills<br>-Students favored the increased integration of digital media	-More in-depth learning of bedside examination skills<br>-Students favored the increased integration of digital media
7	Fog-Petersen, Borgnakke, Hemmingsen, Gefke, and Arnfred (2020)	Clerkship students' use of a video library for training the mental status examination	Medicine ; 4 students<br>**Denmark**	Ethnographic method of participant observation	To explore the students' use of a video library to compensate for the limited amount of contact with patients during their clerkships	Students' autonomous and arbitrary use of the video library.<br>Scheduled their video sessions according to their individual needs.<br>Students blended experiences gained from the library and in the ward<br>Isolating the tasks within the examination process, allowed students to easily transfer their learning between the two settings	**Technology as an adjunct**<br>Beneficial blended learning within the clerkship<br>Divided portions of PE- focus on observable signs and symptoms
8	Mai, Woo, Boles, and Jetty (2018)	Point-of-Care Ultrasound Performed by a Medical Student Compared to Physical Examination by Vascular Surgeons in the Detection of Abdominal Aortic Aneurysms	Medicine; 1 student, 64 patients<br>**Canada POCUS**	a prospective, observer-blinded study	To determine the test characteristics of point-of-care ultrasonography performed by a medical student versus physical examination by vascular surgeons compared to a gold standard reference scan for the detection of AAAs	Point-of-care ultrasonography performed by a medical student is highly accurate and more effective in detecting AAAs than physical examination by vascular surgeons.<br>Opportunity for POCUS training at the medical student level as an extension to physical examination may lead	**Point of care**<br>more effective in detecting AAAs than physical examination by vascular surgeons

						to improved detection of AAAs.	
9	Lehmann, Seitz, Bosse, Lutz, and Huwendiek (2016)	Student perceptions of a video-based blended learning approach for improving pediatric physical examination skills	Medicine; 299<br>4th year students<br>**Germany**	Post intervention mixed method 10-item survey	To determine whether a video-based blended learning approach is well accepted and perceived as being effective for real pediatric patient encounters at the bedside	Preparation with instructional videos combined with bedside teaching, were perceived to improve the acquisition of pediatric physical examination skills.<br>Strong agreement that videos are beneficial for teaching the **practical process** of the physical examination in children	**Technology as an adjunct**<br>Improved student confidence<br>Perceived improvement in skill acquisition
10	Deleo and Geraghty (2018)	iMidwife: midwifery students' use of smartphone technology as a mediated educational tool in clinical environments	Midwifery; 29 students<br>**Australia**	10 question self-administered online survey was used to collect data	This study aimed to define the impact of smartphones in clinical decision-making and learning whilst in clinical areas, by midwifery students at the point of care	- Smartphones enhance the learning and mobility of resources in workplace environments<br>Benefits include; timely access to information to understand complex conditions and to support decision-making; assist with breastfeeding education; to clarify blood results	Smartphone<br>accessible information that supported decision-making<br>Timely access
11	O'Connor and Andrews (2016)	Using Co-Design with Nursing Students to Create Educational Apps for Clinical Training	Nursing; 20<br>final year students<br>**Ireland**	Socio-cognitive engineering approach using co-design process	The aim of this study is to explore the co-design process when creating a clinical skills based smartphone app and identify the features and functions nursing students need in a personalised	Mobile devices and educational apps are used within socio-cultural systems<br>Student desire for step-by-step instructions for performing clinical skills, and information to support use of specialised pieces of equipment and more advanced techniques	Smartphone<br>Student embrace of app based clinical educational tools

					educational app		
12	Mather, Cummings, and Gale (2018)	Mobile Learning in Nursing:<br><br>Tales from the Profession	Nursing; **Australia**	Focus groups questionnaire and interviews	To understand the mobile learning paradox of easy, timely, and convenient access to health information being more available away from the workplace, rather than when at work	A lack of governance to support use of mobile technology<br><br>Potential for mobile technology and mobile learning to contribute to improving patient outcomes and enhancing student learning.	Smartphone<br><br>Governance barriers<br><br>*no comment regarding PE specifically (bedside learning)
13	Mather, Cummings, and Allen (2014)	Nurses' use of mobile devices to access information in health care environments in Australia: a survey of undergraduate students. JMIR mHealth and uHealth	Nursing; 84 students;<br><br>**Australia**	Survey	To identify differences in behavior of undergraduate nurses in accessing information, using a portable or mobile device, when undertaking WIL compared to other non-work situations.	Work-related mobile learning is being undertaken, in situ, by the next generation of nurses who expect easy access to mobile or portable devices at the workplace, to ensure safe and competent care is delivered to their patients.	Mobile device<br><br>*no comment regarding PE specifically – (bedside learning)
14	Rashid-Doubell, Mohamed, Elmusharaf, and O'Neill (2016)	A balancing act: a phenomenological exploration of medical students' experiences of using mobile devices in the clinical setting	Medicine; Final year students<br><br>**The Kingdom of Bahrain**.	Interpretative phenomenolo gy	To describe the experiences of senior students using mobile devices in a clinical setting while learning and interacting with clinical teachers, patients and each other, and to identify challenges that facilitated or impeded the use of such devices in the hospital	The findings showed that using mobile devices in the clinical area as a learning tool was not a formalised process. Rather, it was opportunistic learning at the bedside<br><br>Students used their devices to gather information rapidly. This information gathering facilitated learning about a clinical topic at the bedside	Smartphone<br><br>Rapid/timely access<br><br>Facilitated learning about a clinical topic at the bedside

15	Gavali, Khismatrao, Gavali, and Patil (2017)	Smartphone, the New Learning Aid amongst Medical Students	Medicine 446 students<br><br>**India**	observational cross-sectional study	To evaluate the use of smartphones as an educational tool amongst the medical students. The study also aimed at identifying the common medical application used by the students	Smartphone use amongst medical students as learning aid for various medical applications is rapidly advancing<br><br>90% students, reported to have technological skills to use smartphones, for medical education, communication, and instant access during bedside teaching	Smartphone<br><br>Timely access to information<br><br>Student support
16	Friederichs, Marschall, and Weissenstein (2014)	Practicing evidence based medicine at the bedside: a randomised controlled pilot study in undergraduate medical students assessing the practicality of tablets, smartphones, and computers in clinical life	Medicine 120 third year students<br><br>**Germany**	Randomised controlled pilot study<br><br>Questionnaire design	To determine possible differences between using smartphones, tablets, and stationary computers as search devices at the bedside	Use of mobile phone devices at the bedside is being accepted by students, - sufficient technical skills to perform a literature search at the bedside.<br><br>Convenient for rapid access at the bedside	Smartphone<br><br>Support decision making<br><br>Rapid access
17	Baccin, Dal Sasso, Paixão, and de Sousa (2020)	Mobile Application as a Learning Aid for Nurses and Nursing Students to Identify and Care for Stroke Patients Pretest and	Nursing 115 nurses and 35 nursing students **Brazil**	A quantitative quasi-experimental study	The study aimed at measuring the knowledge of nurses and nursing students in the detection and care of cerebrovascular accident	A significant knowledge gain after the use of the app, particularly in terms of the detection scales and interpretation of the imaging tests.<br><br>The mSmartAVC app used at	Mobile App device<br><br>Support decision making

		Posttest Results				the bedside supported decision making for detection and nursing care.	
18	Higgins, Iu, Carr, Ware, and Van Zundert (2021)	Techniques to select site of insertion for a peripheral intravenous catheter with vessel locating devices using light, sounds or tactile actions (or palpations).	Nursing; 32 novice nurses<br>**Australia**	Inter- subject incomplete counterbalanced research design	To assess if the choice of PIVC insertion site by a novice clinician compared to an expert can be improved with the use of vein locating technology	Vessel locating devices do not enhance the ability of novice clinicians any greater than palpation when selecting suitable forearm veins<br>May benefit expert clinicians find vessels<br>Novice need more skills in manual palpation	Point of care<br>Infrared not beneficial for novice<br>Greater emphasis on manual palpation required for novice nurses.
19	Fox, Lahham (30)	Hypertrophic Cardiomyopathy in Youth Athletes: Successful Screening With Point-of-Care Ultrasound by Medical Students	Medicine 1st and 2nd year medical students<br>**USA**	Prospective design	The objective of this study is to determine whether minimally trained medical student volunteers can use ultrasound to screen for HCM	Volunteer medical students, using point-of-care ultrasound, were able to effectively screen for HCM in young athletes.	POCUS<br>Medical students can identify Hypertrophic cardiomyopathy using POCUS
20	Chandra, Laoteppitaks (38)	Zooming- out COVID- 19: Virtual clinical experiences in an emergency medicine clerkship	Medicine 67 senior students<br>**USA**		To explore a novel clinical educational experience for senior medical students in the form of clinical callbacks that provide students the opportunity to interact live with patients	- Beneficial students engagement<br>-Student exposure to appreciate the breadth of chief complaints treated in the ED -opportunity to work through clinical reasoning<br>-Faculty ability to continue to teach students clinically, one-on- one, in a virtual setting that supported learning.	Telemedicine during placement<br>Telemedicine used for follow up assessment

21	Weber, Dua (36)	An outpatient telehealth elective for displaced clinical learners during the COVID-19 pandemic	Medicine Fourth year students<br>**USA**	Opportunistic design to explore a novel intervention	To provide students with opportunities to utilise telemedicine technologies for authentic patient encounters and enable them to understand telehealth applications and factors affecting the ability of patients to connect remotely	Students identified clinical educational value, citing opportunities to develop patient management plans consistent with in-person experiences.<br>-Vital signs were collected via personal home monitoring for PE	Telehealth placement<br>Vital signs were collected via personal home monitoring
22	Rupley, Grilo (37)	Mobilisation of health professions students during the COVID-19 pandemic	Interprofessional (Medicine, Nursing ,Public Health, Social work) **USA**	Narrative report-	A service-learning framework can harness students in roles that support critical patient-focused initiatives while also allowing for continuing education through students participation.	Student training in virtual care and use of EMR remotely required<br>Blood pressure cuff required for vitals	Telemedicine placement
23	Cain, Shafer Klink (39)	Telemedicine implementation in family medicine: Undergraduate clerkship during COVID- 19 pandemic	Medicine **USA**	Pilot intervention. 5 item Likert scale survey	Exploration of virtual visits with patients as an alternative to real- time patient contact during the COVID- 19 crisis	Meaningful connection with the patient was established and was easy and intuitive<br>Student felt confident in ability to thoroughly assess patient's complaints in virtual setting - preceptor was able to teach sufficiently using virtual format<br>Physical examination options were limited in this format.	Telemedicine placement
24	Bove and Carroll (34)	Comparing Traditional Blood Pressure with a	Nursing; 51 final year	Descriptive study	To compare blood pressure measure ments generated from	Findings suggest that there are differences in the mean measures between the two	Mobile device app<br>Lack of accuracy

		Mobile Health Application	students<br>**USA**	Convenience sampling	a traditional automated b lood pressure cuff and an Apple iOS application to examine the accuracy of a mobile health applicati on.<br>To assess nursing students' challenges to using mobile health appli cations with their patients.	methods for both systolic blood pressure and diastolic blood pressure.<br>Further, the results indicate that the mobile method tends to overestimate both measures	compared with traditional cuff
25	Goldsworth y, Gomes (42)	Do basic auscultation skills need to be resuscitated? A new strategy for improving competency among nursing students.	Nursing; 127 students<br>**Canada**	The study was a pretest/ posttest randomised design.	The purpose of this study was to explore a specific virtual auscultation learning strategy among undergraduate nursing students and the impact on competency in auscultation of heart and lung sounds versus traditional methods of auscultation education.	The virtual auscultation teaching strategy had a positive impact on increasing competence in cardiac murmur recognition among undergraduate nursing student<br>The virtual auscultation was cost efficient, authentic (real patient sounds versus synthetically derived)	Adjunct
26	Ho, Critchley (29)	Introducing Final-Year Medical Students to Pocket-Sized Ultrasound Imaging: Teaching Transthoracic Echocardiography on a 2-Week Anesthesia Rotation	Medicine 133 final-year students<br>**Hong Kong**	Mixed methods Comparative performance analysis and qualitative feedback	Ultrasound into the final-year medical curriculum by introducing handheld transthoracic echocardiography as part of perioperative assessment.	Most of our final-year students learned the skills needed to acquire the four main TTE views and provided some interpretation of their findings, including use of color Doppler to identify valvulopathy, albeit under supervision from a tutor	POCUS

27	Barnes, Duffy (33)	The Mersey Burns App: evolving a model of validation	Medicine; 42 final year students<br>**United Kingdom**	Mixed methods	To address this issue by systematically evaluating both the reliability of the app and its clinical applicability.	Mersey Burns App can facilitate quicker and more accurate calculations than Lund and Browder charts. Students also preferred the App.<br>Student study showed no difference in mean TBSA estimations (p=0.7).<br>Mean time to completion of calculations was faster and calculations were more likely to be correct with the App (p<0.001).<br>Students favoured the App in emergency setting, confidence in output, accuracy, speed, ease of calculation, overall use and shading (p<0.0001).	Mobile App
28	O'Connor and Andrews 2018	Smartphones and mobile applications (apps) in clinical nursing education: A student perspective	Nursing; 200 students (all years) **Ireland**<br>Mobile App	a descriptive cross-sectional design,<br>Convenience sampling	The aim of this study is to understand the perspectives of nursing students in relation to using smartphones and mobile apps to enhance learning in clinical environments.	Students used a range of educational apps in practice to support learning. The most common of which were a calculator drug reference guide medical dictionary, diseases and disorders handbook, language translator and clinical skills guide	Mobile device app<br>students used a range of educational apps in practice to support learning
29	Pearson et al 2021	Evaluation of Female Pelvic Pain: A Hands-On Simulation to Reinforce Exam	Medical; 650 students<br>USA	Mixed methods including faculty critical action	Not stated	Simulation during clerkship used to learn to perform an appropriate abdominal and pelvic exam<br>This simulation combined	Clinical simulation

		Skills and Introduce Transvaginal Ultrasound	Sim	checklist,student survey, and multiple-choice examination questions		touch-based examination and transvaginal ultrasound assessing for possible reproductive tract pathology<br>foundation that reduced student anxiety when encountering female patients with pelvic symptoms during clinical clerkships	
30	Angarita<br>2019	Improving the competency of medical students in clinical breast examination through a standardised simulation and multimedia-based curriculum	Medicine; 120<br>fourth-year students<br>Colombia<br>Sim	This was a single observation, performance assessment study	This study aimed to determine if Standardized Simulation SMBI training resulted in better performance (examination scores and detection of abnormal findings) than the traditional teaching method	Students exposed to SSMBI curriculum performed better than traditionally-trained students in all domains<br>Significant differences were noted in visual inspection, palpation search pattern and technique, examination of the nipple-areolar complex, and identification and characterisation of a palpable lesion	Clinical simulation
31	Weiner, Morone (44)	E-learning module on chronic low back pain in older adults: evidence of effect on medical student objective structured clinical examination performance.	Medicine; 45 final year students<br>**USA**	Random allocation design		This module significantly improved medical student clinical skills in evaluating CLBP.	Technology as an adjunct

32	Hsu, Hsiang (47)	Nursing students' experiences of using a smart phone application for a physical assessm ent course: A qualitative study	Nursing **Taipei**	qualitative descriptive approach with in-depth interview	The aim of this study was to explore the thoughts and experiences of nursing students who received a smart phone, app-based physical assessment educational intervention	Mobile app as an effective learning aid that promoted transferability of the nursing students' knowledge<br><br>Using the APP, the teachers could offer timely guidance to the students.<br><br>Recommendations: mobile learning should continue to be implemented in nursing education with the scenario-based APP with materials with different levels of difficulty in order to cater for nurses' different demands.	Smartphone App
33	Tokunaga, Takamura (43)	Development of the Virtual Physical Assessment Learning Material That Allows the Learners to Check Drug E‹cacy and Early Detection of Adverse Effects through Virtual Experience	Pharmacy1 25 fourth year Pharmacy students<br><br>Japan	Post intervention Survey design		E-learning of the Physical assessment was perceived to be beneficial by the student learners, particularly for learning the PE process, auscultation sounds, using medical equipment, reading, and interpreting data	Technology as an adjunct
34	Wong, Hai (27)	Point-of-care ultrasound augments physical examination learning by undergraduate	Medicine<br><br>210 final year students Hong Kong	Post intervention Survey design	To explore the potential ability of an educational iniative using POCUS to enhance physical examination skill development in final-	50% of students who answered the survey found POCUS useful to correlate findings<br><br>Improved abdominal examination scores	POCUS<br><br>Correlation with physical findings

		medical students.			year medical students		

*Please note that some of the text appearing in this table is verbatim
